# ﻿Review of the wolf spider genus *Halocosa* Azarkina & Trilikauskas, 2019 from China (Araneae, Lycosidae)

**DOI:** 10.3897/zookeys.1218.137275

**Published:** 2024-11-19

**Authors:** Lu-Yu Wang, Muhammad Irfan, Yuri M. Marusik, Zhi-Sheng Zhang

**Affiliations:** 1 Key Laboratory of Eco-environments in Three Gorges Reservoir Region (Ministry of Education), School of Life Sciences, Southwest University, Chongqing 400715, China; 2 College of Plant Protection, Southwest University, Chongqing 400715, China; 3 Institute for Biological Problems of the North RAS, Portovaya Str.18, Magadan 685000, Russia; 4 Department of Zoology & Entomology, University of the Free State, Bloemfontein 9300, South Africa; 5 Altai State University, Lenina Pr., 61, Barnaul, RF-656049, Russia

**Keywords:** Distribution, Evippinae, Lycosinae, morphology, new record, redescription, synonym, taxonomy

## Abstract

The wolf spider genus *Halocosa* Azarkina & Trilikauskas, 2019 from China is reviewed, including two species: *H.cereipes* (L. Koch, 1878) (♂♀) and *H.hatanensis* (Urita, Tang & Song, 1993) (♂♀). Both species are restricted to northern China, with *H.cereipes* recorded from China for the first time. *Halocosajartica* (Urita, Tang & Song, 1993), **syn. nov.** is synonymized with *H.hatanensis* (Urita, Tang & Song, 1993). Detailed species descriptions, along with morphological photos, genitalia illustrations, SEM photos of the bulbs and photos of live specimens are also presented.

## ﻿Introduction

The wolf spider genus *Halocosa* is a small group within the subfamily Lycosinae, currently comprising three species: *H.cereipes* L. Koch, 1878 (generotype), *H.hatanensis* (Urita, Tang & Song, 1993) and *H.jartica* (Urita, Tang & Song, 1993) (WSC 2024) distributed in the Central Palearctic from the southern Ukraine (Azarkina and Trilikauskas 2019) to western Inner Mongolia (Li and Lin 2016). Of these, only the generotype is known for both sexes, and has been illustrated and described in detail, whereas the other two species, *H.hatanensis* and *H.jartica*, are only known for either female or male (WSC 2024).

While examining the materials from Xinjiang, Qinghai, Ningxia and Inner Mongolia, we found numerous female and male specimens that resembled *H.hatanensis* (male) and *H.jartica* (female). We conducted the present review to understand their taxonomic placement.

## ﻿Materials and methods

**Figure 1.**
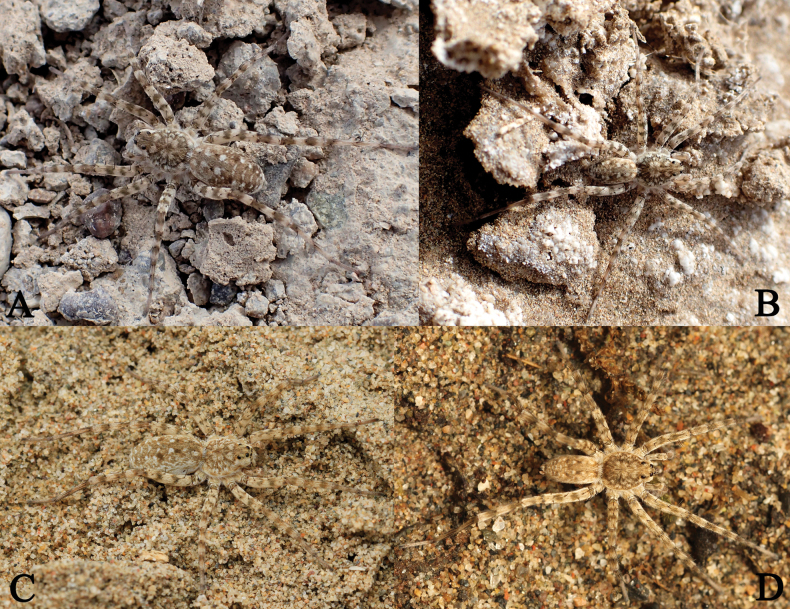
Photos of living specimens **A, B***Halocosacereipes* (L. Koch, 1878) (A female, B male) **C, D***Halocosahatanensis* (Urita, Tang & Song, 1993) (C female, D Same, male).

Photos of living specimens were taken using an Olympus TG3 camera (Fig. 1A, B) and a Canon EOS 7D with an EF 100mm F2.8L lens (Fig. 1C, D). All specimens were preserved in 75% ethanol and examined, illustrated, photographed, and measured using a Leica M205A stereomicroscope equipped with a drawing tube, Leica DFC450 camera, and LAS software (ver. 4.6). Male palps and female epigynes were dissected for examination and illustration. Epigynes were cleared by immersing them in pancreatin (Álvarez-Padilla and Hormiga 2007). Scanning electron microscope (SEM) microphotographs were captured using a Zeiss Evo LS10 SEM. Eye sizes were measured as the maximum dorsal diameter. Leg measurements are provided as: total length (femur, patella and tibia, metatarsus, tarsus). All measurements are in millimeters. Specimens examined here are deposited in the Collection of Spiders, School of Life Sciences, Southwest University, Chongqing, China (SWUC).

Terminology follows Wang et al. (2015), except the word ‘subspermathecae’, which refers to a process attached on the lateral side of spermathecae.

Abbreviations used in the text: ALE, anterior lateral eye; AME, anterior median eye; PLE, posterior lateral eye; PME, posterior median eye.

## ﻿Taxonomy

### ﻿Family Lycosidae Sundevall, 1833

#### ﻿ Halocosa

Taxon classificationAnimaliaAraneaeLycosidae

Azarkina & Trilikauskas, 2019

9209E22E-5BE6-500C-9863-D8C00900C6F1

##### Type species.

*Lycosacereipes* L. Koch, 1878 from Turkmenistan.

##### Diagnosis.

**Figure 2.**
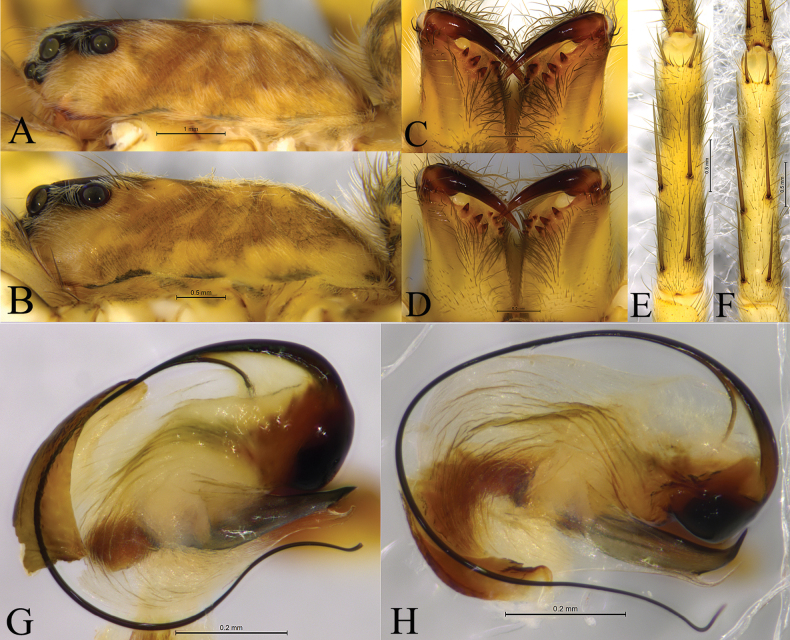
*Halocosa* spp. **A, C, E, G***Halocosacereipes* (L. Koch, 1878) **B, D, F, H***Halocosahatanensis* (Urita, Tang & Song, 1993) **A, B** carapace, lateral view **C, D** chelicerae, ventral view **E, F** tibia of leg I, ventral view **G, H** terminal apophysis and embolus, ventral view.

**Figure 3.**
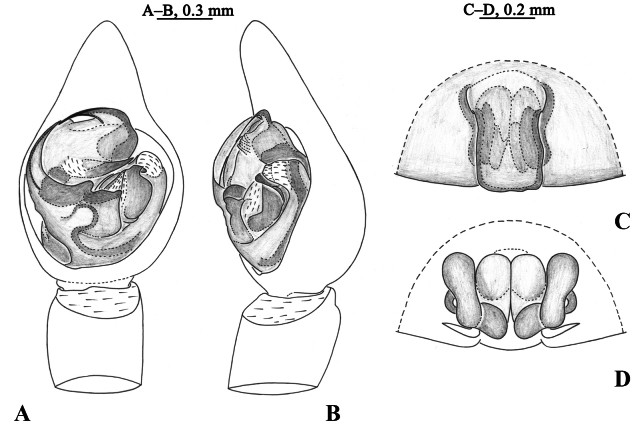
*Halocosacereipes* (L. Koch, 1878) **A** left male palp, ventral view **B** same, retrolateral view **C** epigyne, ventral view **D** vulva, dorsal view.

**Figure 4.**
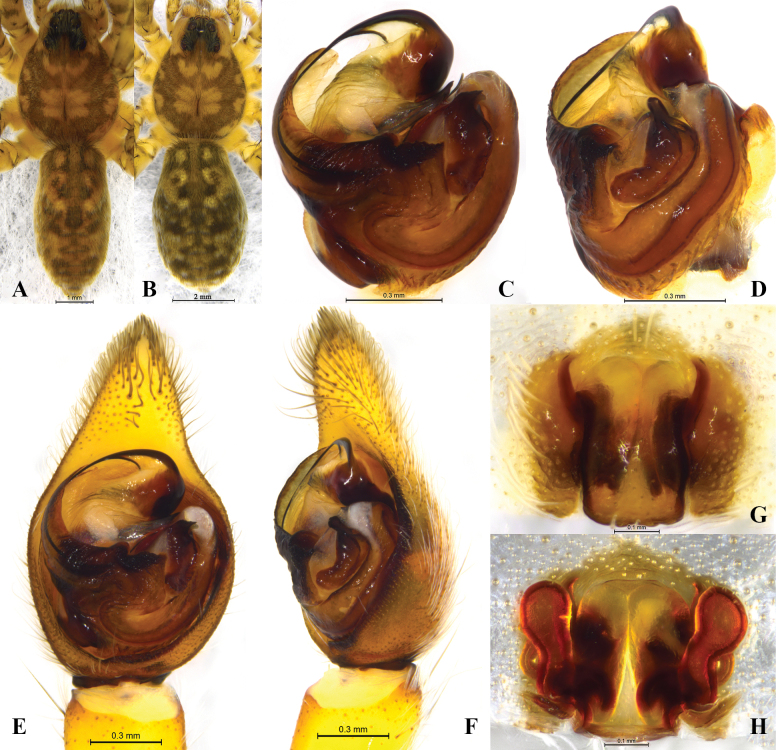
*Halocosacereipes* (L. Koch, 1878) **A** male habitus, dorsal view **B** female habitus, dorsal view **C** left male palp, bulb, ventral view **D** same, retrolateral view **E** left male palp, ventral view **F** same, retrolateral view **G** epigyne, ventral view **H** vulva, dorsal view.

**Figure 5.**
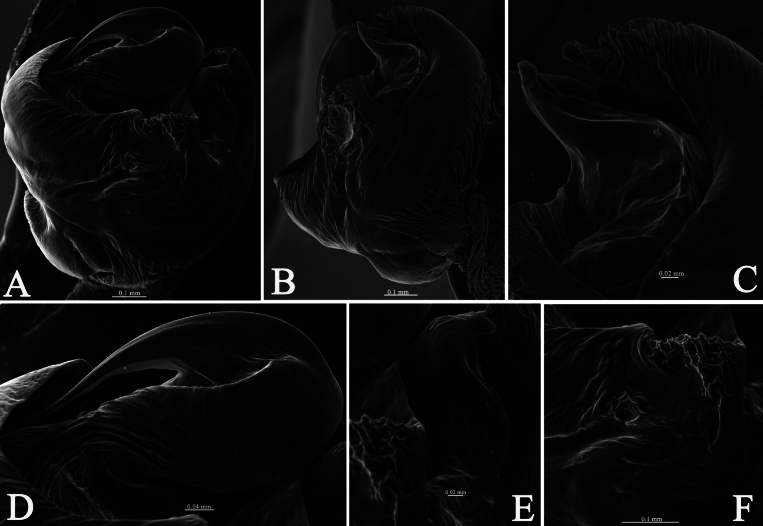
*Halocosacereipes* (L. Koch, 1878) **A** left male pedipalp, bulb, ventral view **B** same, retrolateral view **C** median apophysis and conductor, retrolateral view **D** embolic base, ventral view **E** median apophysis, ventral view **F** tegular sclerite, ventral view.

**Figure 6.**
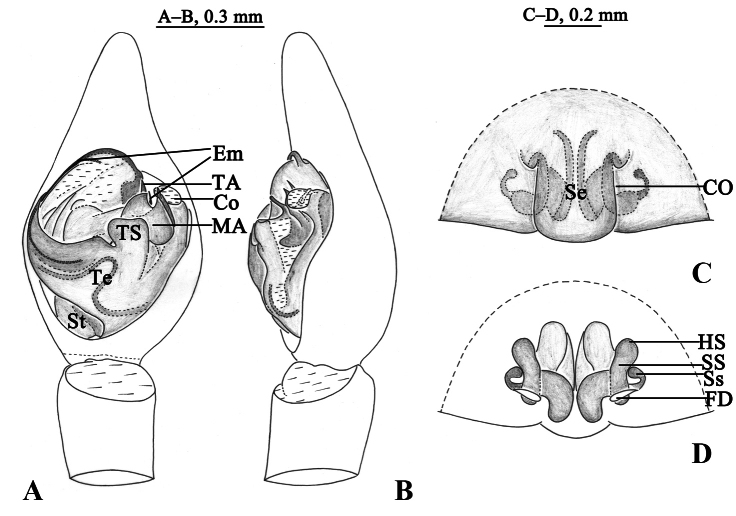
*Halocosahatanensis* (Urita, Tang & Song, 1993) **A** left male pedipalp, ventral view **B** same, retrolateral view **C** epigyne, ventral view **D** same, dorsal view. Abbreviations: CO = copulatory opening; Co = conductor; Em = embolus; FD = fertilization duct; HS = head of spermatheca; MA = median apophysis; Se = septum; SS = stalk of spermatheca; St = subtegulum; TA = terminal apophysis; Te = tegulum; Ts = tegular sclerite.

**Figure 7.**
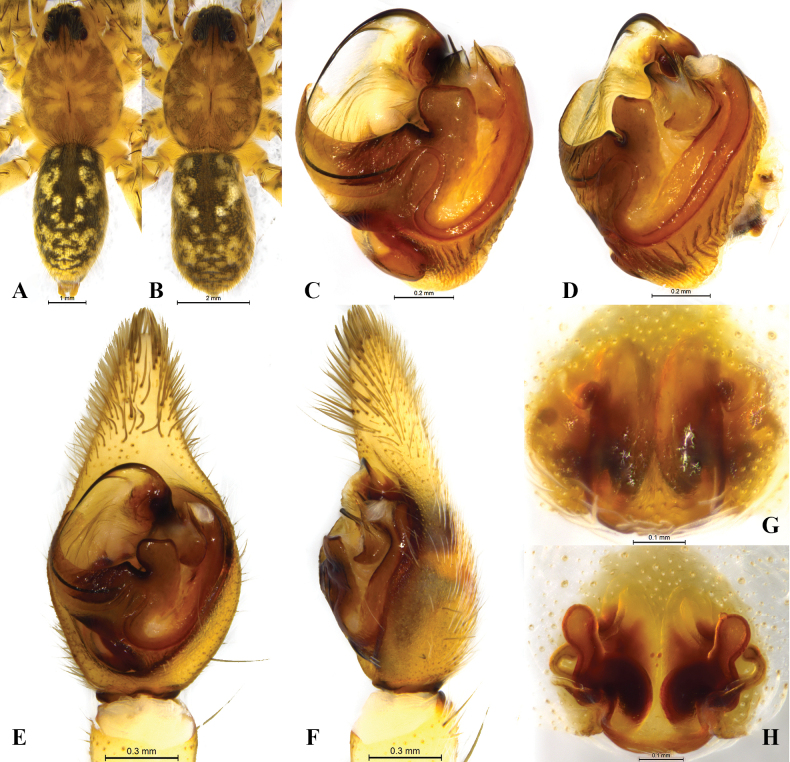
*Halocosahatanensis* (Urita, Tang & Song, 1993) **A** male habitus, dorsal view **B** female habitus, dorsal view **C** left male palp, bulb, ventral view **D** same, retrolateral view **E** left male palp, ventral view **F** same, retrolateral view **G** epigyne, ventral view **H** vulva, dorsal view.

**Figure 8.**
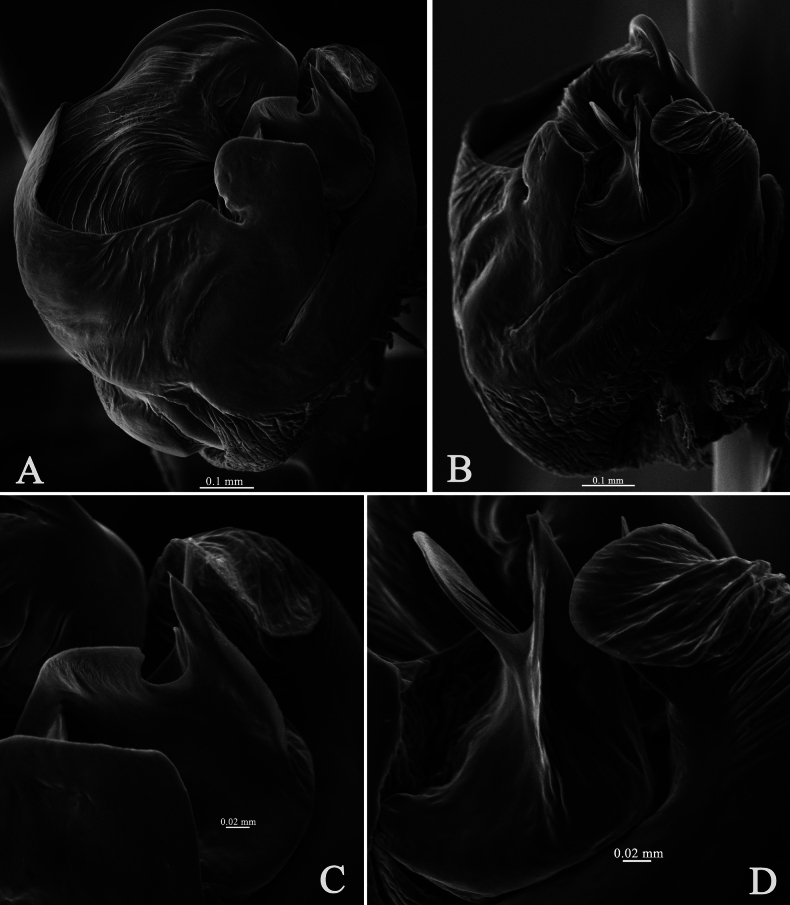
*Halocosahatanensis* (Urita, Tang & Song, 1993) **A** left male pedipalp, bulb, ventral view **B** same, retrolateral view **C** median apophysis, ventral view **D** median apophysis and conductor, retrolateral view.

This genus resembles *Xerolycosa* Dahl, 1908, another genus within subfamily Evippinae. Species of both genera lack a transverse depression on the carapace (Fig. 2A, B), tibia I and II with three pairs of ventral spines (Fig. 2E, F), and male palps with a bifid terminal apophysis (Fig. 2G, H). *Halocosa* can be distinguished by the presence of three retromarginal cheliceral teeth in *Halocosa* (Fig. 2C, D; vs. with two teeth in *Xerolycosa*); embolus lacking accompanied membrane (Fig. 2C, D; vs. present in *Xerolycosa*); strong or small tegular sclerite, bifid terminal apophysis (anterior arm strong and sclerotized, posterior arm thin and membranous) in *Halocosa* (Figs 3A, B, 4C–F, 5, 6A, B, 7E, F, 8; vs. both are membranous in *Xerolycosa*); wide square or rectangular septum covering whole atrium in *Halocosa* (vs. pear-shaped, partly covering atrium in *Xerolycosa*); slit-like copulatory openings, presence of accessory tube-like glands in *Halocosa* (Figs 3C, D, 4G, H, 6C, D, 7G, H; vs. glands absent in *Xerolycosa*).

##### Description.

Medium sized (6.4–13.21) (Azarkina and Trilikauskas 2019) light colored. Carapace dirty brown with pattern formed by yellowish spots: butterfly like spot around fovea, pair of bean-shaped spots posteriorly from eye field and three pairs of marginal round spots; sternum brown; dorsum of abdomen with variegated pattern formed by numerous paired and unpaired spots on dirty brown background, venter uniformly yellow. Carapace very low (length/height ratio c. 4), furrow between cephalic and thoracic parts absent. Chelicera with three pro- and three retromarginal teeth. Leg formula: 4123 or 4132. Femora I–IV with three dorsal spines, patella with one dorsal, tibia and metatarsi III and IV with two dorsal spines (not indicated in tables). Within the intraspecific, the dorsal spines on tibia and metatarsus can be strong or weak, almost indistinguishable from large setae.

Palp with droplet-shaped cymbium, subtegulum (*St*) small, placed on prolateral side; tegulum large, going rather high on prolateral side with long ridge (*Tr*) on prolateral side that hold and hide part of embolus (*Em*); retrolateral part of tegulum with extension directed anteriorly, terminating by conductor, median part with tegular sclerite (*TS*); seminal duct thin, with “sharp” loop (*Sl*) on prolateral half; median apophysis located closer to retrolateral part of tegulum, without extensions, inner side of median apophysis with kind of pocket (or furrow) (*MA*), that holds tip of embolus and seems to serve as functional conductor; in retrolateral view median apophysis concave; embolic division with large sharply pointed terminal apophysis (*TA*) accompanied by membranous subterminal apophysis (*SA*); embolus long whip-like, smoothly rounded, slightly bent near tip, making almost whole circle, partly hidden by tegular ridge and median apophysis, base of embolus located in position of 2 o’clock.

Epigyne relatively small, one-fifth the width of the abdomen, densely covered with white setae to such extent that adult female could be considered as juvenile, especially in the field; fovea/atrium absent, totally covered with rectangular septum, septal stem absent, copulatory openings located in anterior part of epigynal plate, open into deep bulge which turns to wide, weakly sclerotized duct going straight down, near epigastral fold this duct turns up into strongly sclerotized, partly twisted, duct terminating by more or less clavate spermatheca; heavily sclerotized part of duct with finger-like or clavate accessorial gland (*Ag*).

##### Relationships.

Azarkina and Trilikauskas (2019) placed this genus in Lycosinae due to “the latero-median origin of the embolus that is situated in a shallow and wide depression”. This placement appears to be incorrect. All Lycosinae have palea (lacking in *Halocosa*), the median apophysis originates prolaterally and stretches horizontally (vs. originates retrolaterally and stretches parallel to cymbium axis), and the septum is not covered with setae (with exception of *Arctosa*) (vs. covered with setae). In addition, the carapace in *Halocosa* is very low, 3–4 times longer than high in comparison to Lycosinae (c. 2.4 longer than wide). To the best of our knowledge, the copulatory organs of this genus, as well as the flattened carapace, fit well with those known in Evippinae. Therefore, we consider *Halocosa* in Evippinae.

##### Composition.

Two species: *H.cereipes* (L. Koch, 1878) and *H.hatanensis* (Urita, Tang & Song, 1993).

##### Biology.

*Halocosacereipes* is a dweller of saline places. It was collected around salt lakes (Iran, Azerbaijan, around the Aral Sea), and on the low seashore in the Crimea (personal data). The same habitats were reported by Azarkina and Trilikauskas (2019). Numerous spots on the carapace and abdomen, and leg annulation make the spiders very cryptic, and invisible if they are not moving.

##### Distribution.

From southern Ukraine to western Inner Mongolia, south to Iran (Azarkina and Trilikauskas 2019; Nentwig et al. 2021). In China, known from Xinjiang, Ningxia, Qinghai and Inner Mongolia (present paper).

#### ﻿ Halocosa cereipes

Taxon classificationAnimaliaAraneaeLycosidae

(L. Koch, 1878)

CB1DDB3D-DCBA-5A4D-BC30-55E4B7CB7752

**Figure 9.**
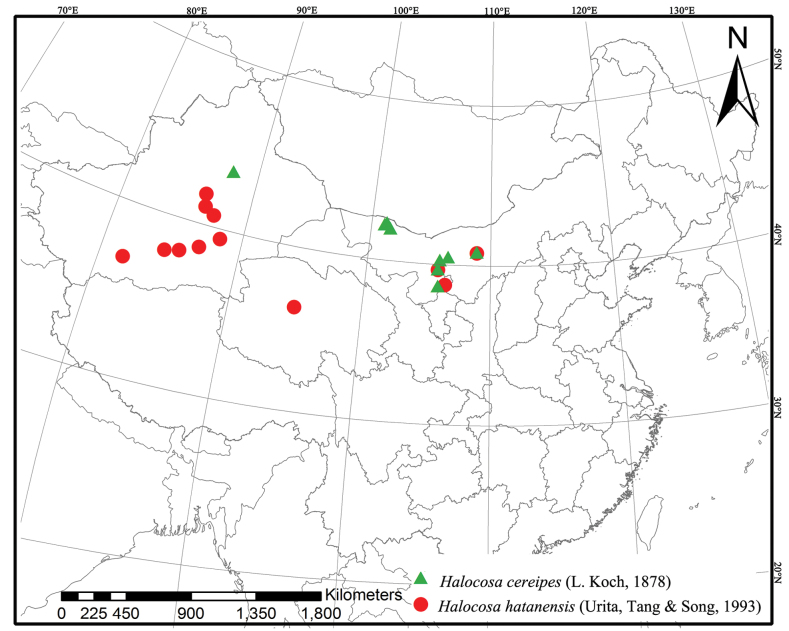
Distribution of *Halocosa* in China.

Figs 1A, B , 2A, C, E, G , 3 , 4 , 5 , 9 (角盐狼蛛)

Lycosa cereipes L. Koch, 1878: 68, pl. 2, fig. 6 (♂).
Pirata cereipes : Roewer 1955: 283.
Evippa apsheronica Marusik, Guseinov & Koponen, 2003: 52, figs 1–3 (♀); Ponomarev and Tsvetkov 2004: 86, figs 1–2 (♂♀).
Halocosa cereipes : Azarkina and Trilikauskas 2019: 557, figs 1–8, 15–18, 26–69 (♂♀, designated the neotype from the type locality).

##### Material examined.

China: **Xinjiang**: 1♀, Urumchi, Chaiwopu, 43°31.834′N, 087°53.695′E, 1097 m, 22 April 2014, L.Y. Wang & X.W. Meng leg. **Inner Mongolia**: 2♂ 2♀, Ejinaqi, Tiane Lake, 42°00.671′N, 101°35.242′E, 890 m, 5 June 2015, T. Lu and G.Q. Huang leg. • 3♂ 2♀, Ejinaqi, Juyanhai, 42°13.729′N, 101°04.404′E, 906 m, 5 June 2015, T. Lu and G.Q. Huang leg. • 9♂ 7♀, Ejinaqi, Juyanhai, 42°20.273′N, 101°15.020′E, 895 m, 5 June 2015, T. Lu and G.Q. Huang leg. • 4♂ 3♀, Alashanzuoqi, Jilantai Salt Lake, 39°43.281′N, 105°44.705′E, 1017 m, 7 June 2015, T. Lu and G.Q. Huang leg. • 10♂ 10♀, Alashanzuoqi, Jilantai, Dongshawo, 39°44.399′N, 105°46.484′E, elev. 1024 m, 7 June 2015, T. Lu and G.Q. Huang leg. • 1♂, Alashanzuoqi, Helan Mountain, Nan Temple, 38°39.918′N, 105°48.436′E, 1976 m, 9 June 2015, T. Lu and G.Q. Huang leg. • 1♂, Alashanzuoqi, Qinggele, 40°17.051′N, 105°51.200′E, 1165 m, 11 June 2015, T. Lu and G.Q. Huang leg. • 8♂ 17♀, Alashanzuoqi, Liutuan, Dongqing Lake, 40°30.288′N, 106°30.384′E, 1030 m, 11 June 2015, T. Lu and G.Q. Huang leg. • 1♂, Bayannur, Wulateqianqi, Eerdengbulage, Wuliangsuhai, 40°51.577′N, 108°50.906′E, 1025 m, 14 June 2015, T. Lu and G.Q. Huang leg.

##### Diagnosis.

This species is similar to *H.hatanensis* (Figs 2B, D, F, H, 6–8), but differs by the dwarf tegular sclerite vs. large and almost square; the short, strong and flat terminal apophysis vs. long and crooked; the wide and short subterminal apophysis vs. long and thin; median apophysis not bifurcate and the end bent to conductor vs. bifurcate, ventral branches curved, dorsal branch straight and pointed (Figs 2G, 3A, B, 4C–F, 5); the arc-shaped copulatory openings, located on the anterior position of the septum vs. located below the septum; and the width of spermathecal head greater than the width of spermathecal stalk vs. width of spermathecal head almost the same as the width of spermathecal stalk (Figs 3C, D, 4G, H).

##### Redescription.

Males total length 7.65–10.19. One male (Figs 1B, 4A, from Dongqing Lake) total length 7.65, carapace 3.72 long, 3.69 wide; opisthosoma 4.11 long, 2.01 wide. Eye sizes and interdistances: AME 0.20, ALE 0.14, PME 0.35, PLE 0.33; AME–AME 0.10, AME–ALE 0.05, PME–PME 0.26, PME–PLE 0.31. Clypeus height 0.17. Leg measurements: I 11.68 (3.13, 3.93, 2.72, 1.90); II 10.62 (2.79, 3.47, 2.57, 1.79); III 10.53 (2.78, 3.15, 2.94, 1.66); IV 14.69 (3.69, 4.33, 4.44, 2.23).

***Palp*** (Figs 2G, 3A, B, 4C–F, 5). Tip of cymbium 3 times shorter than cymbium; length/width ratio c. 1.7. The end of terminal apophysis short, strong and flat, subterminal apophysis membranous, as long as terminal apophysis. Median apophysis vertical, concave in lateral view. Tegular sclerite strongly sclerotized and dwarf. Embolus long and whip-like, smoothly rounded, slightly bent near tip, it makes almost whole circle, partly hidden by tegular ridge and median apophysis, base of embolus located in position of 2 o’clock. Conductor membranous.

**Females** total length 6.59–13.18. One female (Figs 1A, 4B, from Dongqing Lake) total length 6.59, prosoma 3.59 long, 2.47 wide; opisthosoma 2.99 long, 2.00 wide. Eye sizes and interdistances: AME 0.22, ALE 0.17, PME 0.37, PLE 0.32; AME–AME 0.12, AME–ALE 0.05, PME–PME 0.31, PME–PLE 0.35. Clypeus height 0.14. Leg measurements: I 11.41 (3.30, 3.86, 2.45, 1.80); II 10.84 (3.03, 3.63, 2.51, 1.67); III 10.76 (3.06, 3.16, 2.72, 1.82); IV 15.26 (4.09, 4.50, 4.50, 2.17).

***Epigyne*** (Figs 3C, D, 4G, H). Septum 1.7 times longer than wide. Copulatory openings arc-shaped, located on the anterior position of the septum. Spermathecal heads slightly inflated, approaching to the anterior margins of spermathecal stalks. Spermathecal stalks as wide as heads. Accessorial gland arc-shaped, with a small and spherical head. Fertilization ducts hook-shaped.

##### Distribution.

China (Xinjiang and Inner Mongolia, new records) (Fig. 9), southern Ukraine, northern Caucasus to southern part of West Siberia, Azerbaijan, Iran, Kazakhstan, Turkmenistan and Tajikistan.

#### ﻿ Halocosa hatanensis

Taxon classificationAnimaliaAraneaeLycosidae

(Urita, Tang & Song, 1993)

4D65EAAA-A9B4-54D0-B433-995B2131CB2D

Figs 1C, D , 2B, D, F, H , 6 , 7 , 8 , 9 (哈腾盐狼蛛)

Pardosa hatanensis Urita, Tang & Song, 1993: 46, figs 1A, B (holotype ♂ from Hatan Tohoi, Bayannur Meng, Inner Mongolia, China, deposited in Inner Magnolia Normal University, Hohhot, China, not examined).
Halocosa hatanensis : Azarkina and Trilikauskas 2019: 557 (transferred from Pardosa).
Pardosa jartica Urita, Tang & Song, 1993: 47, figs 2A, B (holotype ♀ from Jartai, Alxa Meng, Inner Mongolia China, deposited in Inner Magnolia Normal University, not examined). syn. nov.
Halocosa jartica : Azarkina and Trilikauskas 2019: 557 (transferred from Pardosa).

##### Material examined.

China: **Xinjiang**: 1♀, Qiemo County, 31 July 2006, F. Zhang leg. • 1♂ 2♀, Korla City, Tashidian Town, 25 May 2009, D. Sun and Y.W. Zhao leg. • 2♀, Ruoqiang County, Taitema Lake, 39°28.309′N, 88°16.791′E, 789 m, 10 May 2013, L.Y. Wang leg. • 1♂, Qiemo County, 38°41.504′N, 86°53.235′E, 1029 m, 10 May 2013, L.Y. Wang leg. • 1♀, Yuli County, 41°06.476′N, 86°30.650′ E, 868 m, 1 June 2014, L.Y. Wang and X.K. Jiang leg. • 7♂ 4♀, Yuli County, 40°43.939′N, 87°20.139′E, 863 m, 1 June 2014, L.Y. Wang and X.K. Jiang leg. • 1♂ 10♀, Ruoqiang County, Taitema Lake, 39°28.309′N, 88°16.791′E, 789 m, 1 June 2014, L.Y. Wang and X.K. Jiang leg. • 3♂ 2♀, Qiemo County, 38°41.536′N, 86°53.263′E, 1004 m, 2 June 2014, L.Y. Wang and X.K. Jiang leg. • 6♂, Qiemo County, Kalamilan River, 37°57.796′N, 84°26.794′E, 1260 m, 2 June 2014, L.Y. Wang & X.K. Jiang leg. • 1♀, Yutian County, Keliya River, 36°51.678′N, 81°42.622′E, 1382 m, 3 June 2014, L.Y. Wang and X.K. Jiang leg. **Qinghai**: 1♂, Geermu, 15 Septemper 2002, M.S. Zhu leg. **Inner Mongolia**: 3♂ 1♀, Alashanzuoqi, Jilantai (Jartai) Salt Lake, 39°43.281′N, 105°44.705′E, 1017 m, 7 June 2015, T. Lu and G.Q. Huang leg. • 1♂ 2♀, Bayannur, Wulateqianqi, Eerdengbulage, Wuliangsuhai, 40°51.577′N, 108°50.906′E, 1025 m, 14 June 2015, T. Lu and G.Q. Huang leg. **Ningxia**: 1♂ 1♀, Shizuishan City, Pingluo County, Sha Lake, 38°47.890′N, 106°20.934′E, 1104 m, L.Y. Wang, H.Y. Liu and K. Yu leg.

##### Diagnosis.

This species is similar to *H.cereipes* (L. Koch, 1878) (Figs 2A, C, E, G, 3–5), but differs by the tall and almost square tegular sclerite vs. dwarf in *H.cereipes*; the long and crooked terminal apophysis vs. short, strong and flat; the long and thin subterminal apophysis vs. wide and short; the bifurcate median apophysis, ventral branches curved, dorsal branch straight and pointed vs. not bifurcate, end bent to conductor (Figs 2H, 6A, B, 7C–F, 8); the crack-shape copulatory openings and located below of the septum (vs. arc-shaped, located on the anterior position of the septum); and the width of spermathecal head almost the same as the width of spermathecal stalk (vs. width of spermathecal head greater than the width of spermathecal stalk) (Figs 6C, D, 7G, H).

##### Description.

**Males** total length 7.52–10.36. One male (Figs 1D, 7A, from Qiemo County) total length 7.52: carapace 3.84 long, 2.73 wide; opisthosoma 3.82 long, 2.04 wide. Eye sizes and interdistances: AME 0.20, ALE 0.14, PME 0.36, PLE 0.32; AME–AME 0.13, AME–ALE 0.05, PME–PME 0.24, PME–PLE 0.30. Clypeus height 0.18. Leg measurements: I 11.82 (3.20, 4.03, 2.73, 1.86); II 11.52 (3.18, 3.81, 2.66, 1.86); III 11.58 (3.04, 3.55, 3.24, 1.75); IV 15.84 (4.00, 4.74, 4.83, 2.23).

***Palp*** (Figs 2H, 6A, B, 7C–F, 8). Cymbium c. 1.9 times longer than wide. The end of terminal apophysis curving, subterminal apophysis thin and membranous, shorter than the length of terminal apophysis. Median apophysis vertical and bifurcate, concave in lateral view. Tegular sclerite tall and almost square. Embolus long whip-like, smoothly rounded, slightly bent near tip, making almost whole circle, partly hidden by tegular ridge and median apophysis, base of embolus located in position of 2 o’clock. Conductor membranous.

**Females** total length 8.01–13.21. One female (Figs 1C, 7B, from Qiemo County) total length 8.01: carapace 4.03 long, 2.75 wide; opisthosoma 4.04 long, 2.45 wide. Eye sizes and interdistances: AME 0.23, ALE 0.15, PME 0.35, PLE 0.32; AME–AME 0.10, AME–ALE 0.04, PME–PME 0.27, PME–PLE 0.32. Clypeus height 0.19. Leg measurements: I 10.74 (3.05, 3.66, 2.29, 1.74); II 10.25 (2.88, 3.50, 2.20, 1.67); III 10.60 (2.90, 3.41, 2.63, 1.66); IV 14.62 (3.76, 4.55, 4.30, 2.01).

***Epigyne*** (Figs 6C, D, 7G, H). Copulatory openings crack-shaped and located below of the septum. Spermathecal heads slightly inflated, approaching the anterior margins of spermathecal stalks. Spermathecal stalks as wide as heads. Accessorial gland arc-shaped, with a small and spherical head. Fertilization ducts small, crescent-shaped.

##### Distribution.

China (Xinjiang, Qinghai, Ningxia and Inner Mongolia) (Fig. 9).

##### Remarks.

Sample collected from the type locality of *H.jartica* contains specimens of both sexes. Comparison of these specimens with illustrations and descriptions of the *H.jartica* female and male of *H.hatanensis* reveals no differences, and therefore, we synonymized these names. The distance between the type localities is about 140 km. Although two species were described in the same paper, we consider *H.hatanensis* as the senior synonym because of page priority, and also because males have more diagnostic characters than females.

## Supplementary Material

XML Treatment for Halocosa

XML Treatment for Halocosa cereipes

XML Treatment for Halocosa hatanensis

## ﻿Additional information

### Author ORCIDs

Lu-Yu Wang https://orcid.org/0000-0002-5250-3473

Muhammad Irfan https://orcid.org/0000-0003-0445-9612

Yuri M. Marusik https://orcid.org/0000-0002-4499-5148

Zhi-Sheng Zhang https://orcid.org/0000-0002-9304-1789

### Data availability

All of the data that support the findings of this study are available in the main text.
